# A Detailed Modular Analysis of Heat-Shock Protein Dynamics under Acute and Chronic Stress and Its Implication in Anxiety Disorders

**DOI:** 10.1371/journal.pone.0042958

**Published:** 2012-08-22

**Authors:** K. Sriram, Maria Rodriguez-Fernandez, Francis J. Doyle

**Affiliations:** 1 Institute of Collaborative Biotechnologies, University of California Santa Barbara, Santa Barbara, California, United States of America; 2 Department of Chemical Engineering, University of California Santa Barbara, Santa Barbara, California, United States of America; 3 Indraprastha Institute of Information Technology (IIIT), Delhi, India; Koc University, Turkey

## Abstract

Physiological and psychological stresses cause anxiety disorders such as depression and post-traumatic stress disorder (PTSD) and induce drastic changes at a molecular level in the brain. To counteract this stress, the heat-shock protein (HSP) network plays a vital role in restoring the homeostasis of the system. To study the stress-induced dynamics of heat-shock network, we analyzed three modules of the HSP90 network—namely trimerization reactions, phosphorylation–dephosphorylation reactions, and the conversion of HSP90 from an open to a closed conformation—and constructed a corresponding nonlinear differential equation model based on mass action kinetics laws. The kinetic parameters of the model were obtained through global optimization, and sensitivity analyses revealed that the most sensitive parameters are the kinase and phosphatase that drive the phosphorylation–dephosphorylation reactions. Bifurcation analysis carried out with the estimated kinetic parameters of the model with stress as bifurcation parameter revealed the occurrence of “mushroom”, a type of complex dynamics in which S-shaped and Z-shaped hysteretic bistable forms are present together. We mapped the molecular events responsible for generating the mushroom dynamics under stress and interpreted the occurrence of the S-shaped hysteresis to a normal level of stress, and the Z-shaped hysteresis to the HSP90 variations under acute and chronic stress in the fear conditioned system, and further, we hypothesized that this can be extended to stress-related disorders such as depression and PTSD in humans. Finally, we studied the effect of parameter variations on the mushroom dynamics to get insight about the role of phosphorylation–dephosphorylation parameters in HSP90 network in bringing about complex dynamics such as isolas, where the stable steady states in a bistable system are isolated and separated from each other and not connected by an unstable steady state.

## Introduction

Exposure to stress alters the homeostasis of a system, which then adapts in an attempt to regain normalcy [1]. At a molecular level, these changes are brought about by a cascade of slow and fast reactions that are tightly regulated by feedforward and feedback loops. For example, in the hypothalamus-pituitary-adrenal (HPA) axis of the brain, the production of hormone cortisol during stress is tightly regulated by the HPA's autoregulatory negative feedback mechanisms [2]. One of the molecular networks that is initiated during stress involves protein chaperones [3]. Chaperones are ATP-dependent heat-shock proteins (HSPs) that have molecular weights ranging from 20 to 110 kDa, and take up multiple roles, from the degradation of a misfolded protein to carrying ligand-bound receptors to the nucleus for transcriptional regulation [4]. Among these heat-shock proteins, HSP90 plays a central role in signaling various downstream regulators that include the transport of cortisol to the nucleus. HSP90 protein is produced through a series of reactions that start with the initiation of heat-shock factors (HSFs) and the formation of HSF trimers. Active HSF1 trimers in turn bind to the heat-shock response elements (HSE) to form complexes that undergo hyper–phosphorylation-dephosphorylation (PdP) reactions. Finally, the hyper-phosphorylated complexes lead to the generation of ATP-assisted closed conformation of HSP90 protein with the help of other co-chaperones such as the proteins p23, Huntingtin interacting protein (HIP) and HSP70/HSP90-organizing protein (HOP). This closed HSP90 conformation is either activated to process the substrates, or is bound to the client proteins for further regulation [5]. HSP90 also regulates its own production by binding to both the HSF monomer and oligomers to complete the negative feedback loop [6].

Mathematical modeling of the chaperone network in the context of heat-shock response with temperature as the stress parameter have been carried out by different groups to account for various experimental observations [7]–[10]. However, the dynamical effects of stress on the chaperone network in fear conditioned animal models that have been used as a paradigm for psychiatric disorders such as depression and post-traumatic stress disorder (PTSD) in humans have not been studied. PTSD is an anxiety disorder that results from prolonged exposure to traumatic events. According to the *Diagnostic and Statistical Manual of Mental Disorders* (DSM-IV) criteria, the core symptoms are impaired concentration, emotional numbing, recurrent flashes of traumatic memories, social withdrawal, and hyperarousal [11].

In animals, the development of a fear conditioned model is an important step towards the understanding of PTSD that aims to model the DSM criterion A in humans; i.e., simulating extreme stressful situations that create a sense of threat and helplessness [11]. Fear conditioning is an associative learning paradigm in which subjects associate neutral stimulus with an aversive stimulus that provokes fear and induces a long-lasting behavior and physiological responses like freezing and startle [12]. At a molecular level, one study indicated that under trace fear conditioning (TFC) protocol, many genes involved in protein folding and quality control (transcription factors *Hspa5*, *Hspb1*, *Dnajb4*) were upregulated, and these results validated with RT-PCR, indicated protein folding as an important pathway in fear conditioning paradigm [13].

In humans, at a molecular level, PTSD is strongly linked to the hyperactive HPA axis, and specifically to a high glucocorticoid-receptor (GR) sensitivity due to a low secretion of salivary and blood glucocorticoids (cortisol in humans and corticosterone in mice) during late night until early morning [14]–[19]. A low cortisol level in the HPA axis at one particular time of the day is a common indicator of PTSD that helps to distinguish the normal function from other comorbid disorders such as depression. GR sensitivity is also related to FK506-binding protein (FKBP), a co-chaperone of HSP90 that helps to translocate the cortisol-bound GR to the nucleus to regulate the expression of GR-sensitive genes [20]. Recently, FKBP5 polymorphism was shown to correlate strongly with PTSD, and presently, it is a strong candidate as a biomarker gene responsible for HPA disturbances [21]. FKBP5 was also found to be at a much lower concentration in persons with PTSD who were exposed to the World Trade Center attacks than in persons without PTSD [22]. Therefore, modeling the dynamics of the HSP90 network that regulates both the cortisol in HPA axis and other co-chaperones such as FKBP5 is important to understand the etiology of PTSD at the molecular level.

All these results from various experiments in animal and human models indicate that the study of the heat shock protein pathways is important in understanding psychiatric disorders under various conditions of stress (acute and chronic). Therefore, in this work, we modeled the effect of acute and chronic stress on the dynamics of HSP90 network and extended this to the human model to make predictions about the occurrence of depression, PTSD and the comorbidity. To model the dynamics, we took time series data from HeLa S3 cells to obtain the kinetic constants through parameter estimation by global optimization. We used the estimated parameter values to perform bifurcation analyses and map the variations of HSP90 concentrations under acute and chronic stress. Since the estimated parameter values vary for different runs, we also performed bifurcation analyses for various parameter sets to determine various possible dynamical scenarios under acute and chronic stress.

## Methods

### Assumptions

**Figure 1:**
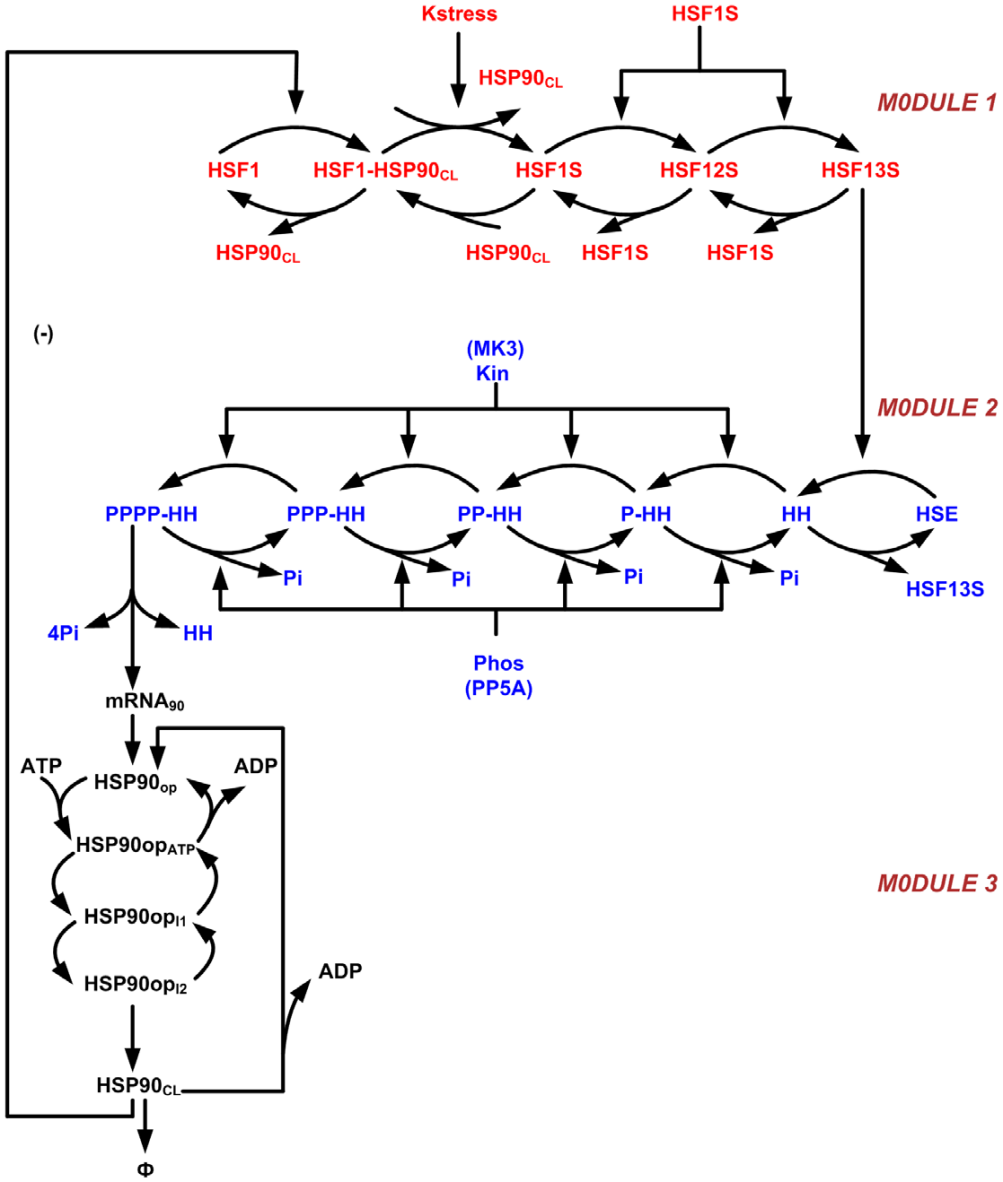
Modular construction of the heat-shock protein 90 (HSP90) network. Three modules form the whole network: HSF1 trimer formation, phosphorylation-dephosphorylation reactions of the HSP90:HSF1 trimer complex, and the conversion of an open to a closed HSP90 protein conformation. Module 1, shown in red, is the trimer formation of HSF1. Inactive HSF1 monomer sequestered by the closed conformation of HSP90 (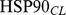) forms a complex, 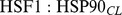. Stress releases the inactive HSF1 monomer from the 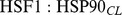 complex, and turns it into an active form, HSF1S, to produce a dimer (HSF12S) slowly, but a trimer (HSF13S) rapidly. The trimer binds to the heat-shock element (HSE) to form a complex, HH, but it is incompetent to transcribe the hsp90 gene, unless strongly phosphorylated. Module 2, shown in blue, consists of the PdP reactions of the complex HH. The trimer HSF1S in the HH complex is hyper-phosphorylated by the kinase (MK3) and dephosphorylated by the phosphatase PP5, a co-chaperone. Hyper-phosphorylated HH (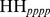) is competent to transcribe the hsp90 gene to produce 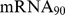, which in turn translates into an open conformation of the HSP90 protein by series of steps. Module 3, shown in black, represents the formation of HSP90 closed conformation in which the open form of 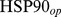 binds to adenosine triphosphate (ATP), and converts it to conformation 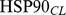 through two step wise intermediate conformations (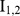). The fate of the closed form, which is competent to bind the substrate or the client proteins is three-fold: it (i) can shed the ATP to attain an open conformer, (ii) can bind to HSF1 monomer to negatively regulate its own production, finally (iii) can degrade to form a dead product, 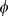.

The HSP90 molecular network, depicted in Fig. 1, has three important modules: (a) formation of HSF1 trimer, (b) phosphorylation-dephosphorylation (PdP) reactions of the complex formed between the active HSF1S trimer and HSE, and (c) conversion of open to closed HSP90 conformation. In constructing these networks, three important assumptions were made: (i) nuclear and cytoplasmic compartments are not separately considered therefore, homogeneity of the cell is assumed, (ii) sufficient numbers of molecules are present for the reactions to take place instantaneously so that stochastic fluctuations are eliminated (see Mizera et al. [23] for stochastic modeling of eukaryotic heat-shock response), (iii) for modules 1 and 2, the kinetic parameters were not known, but experimental time course data from western blots of HeLa cells (human cell lines) were available with temperature as an externally modulated stress parameter [24], [25]. We used this data to find kinetic parameters so that qualitatively similar time profiles were generated. Importantly, for psychiatric disorders such as PTSD and depression, the temperature is constant. However, irrespective of the type of stress involved, the mechanistic steps involved in the production of HSP90 are assumed to be the same. Therefore, the experimental data generated with temperature as a stress parameter is chosen as a starting point to find the kinetic parameters without providing any relationship between the model parameter and temperature, unlike in certain specific heat-shock models [7], [10]. With these three assumptions, we started by building the network in a modular fashion based on mass action kinetic laws. The kinetic equations were either reversible first or second order reactions of the form:
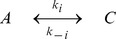
(1)

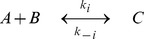
(2)with 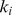 and 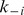 being the forward and backward kinetic constants, respectively. The degradation reactions were always assumed to be first order of the form:
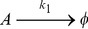
(3)where 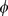 is the degraded form of *A* that is not involved in any other reactions. Because the concentrations of most of the proteins in the HSP90 network were not known, the units of concentration were arbitrarily taken as nM, and the time in seconds.

The PdP reactions were modeled as given in Markevich et al. [26] in the following way. The dual phosphorylation is given by:
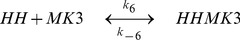
(4)

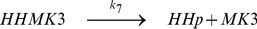
(5)

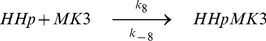
(6)

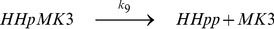
(7)and the dephosphorylation is given by:
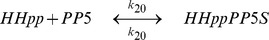
(8)

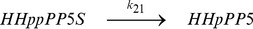
(9)

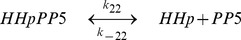
(10)

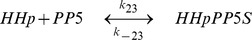
(11)

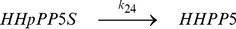
(12)

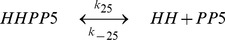
(13)


In the above equations, HH is the HSF13S:HSE complex, 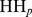, 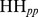, 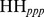, and 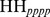 are the mono, di, tri, and tetra-phosphorylated complex, MK3 is the kinase, PP5 is a phosphatase, and PP5S is the activated phosphatase formed during the dephosphorylation. One of the underlying assumptions is that the phosphatase PP5 has a strong binding affinity for HH. Further, PP5 is the phosphatase that has been implicated in the negative regulation of the heat shock factor by preventing its active or hyperphosphorylated state [27] and therefore we assumed that the unphophorylated HH can be bound by phosphatase more strongly than in the phosphorylated state.

Because many reactions are involved in each of the modules, the kinetic steps, kinetic equations, and differential equations are given in the appendices. We used the software program XPPAUT to generate all the bifurcation diagrams, and for numerical integration [28]. We transported the data from XPPAUT to Matlab® to plot the figures. We provide the ordinary differential equation (ODE) file of XPPAUT that we used to simulate the bifurcation diagrams as a separate supplementary file (Model file S1).

## Results

### Construction of HSP90 molecular network modules based on experimental evidence and experimental data

#### Module 1: Formation of the HSF1 trimer

Transcriptional activities of the heat-shock genes are controlled by HSF1 transcription factors that are inert under normal conditions. HSF1 exists as a monomer and sequesters with HSP90 in unstressed cells. Stress dissociates the HSP90:HSF1 complex to release “active” HSF1 monomer (HSF1S) that rapidly forms a homo-dimer (HSF12S) and a homo-trimer (HSF13S) [29], [30]. We assumed that the trimer forms in a step wise manner. HSP90 also sequesters with both the active monomer and oligomers, but this sequestration is not considered in the network. The active trimer then binds to the heat-shock gene promoter HSE to form a complex, but this complex is incompetent to induce transcription [31] until the hyper-phosphorylation reactions in the complex take place.

**Figure 2:**
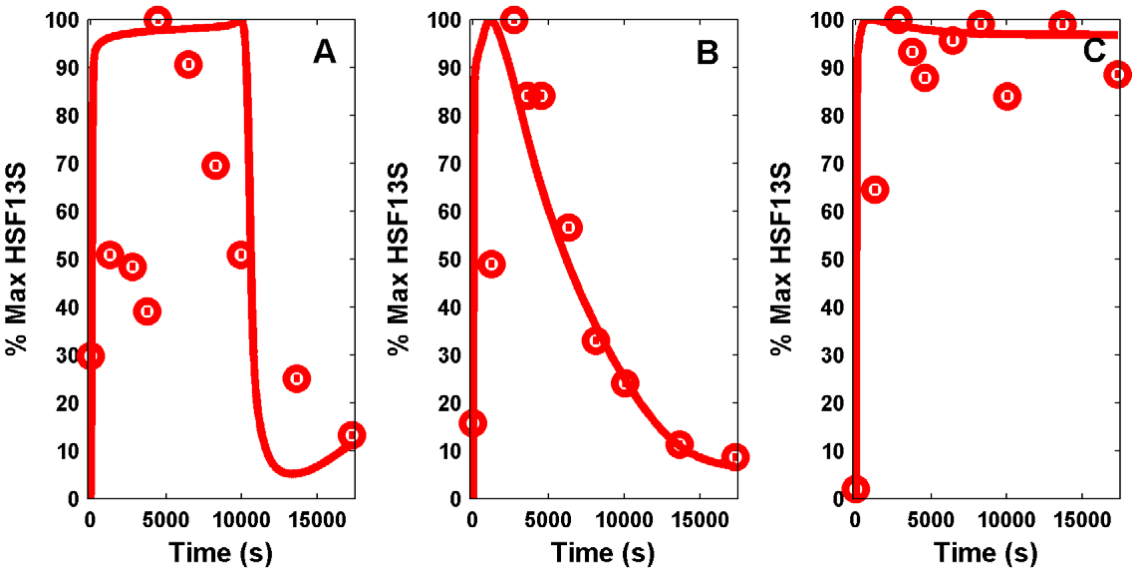
Simulated and experimental time series of heat-shock factor 1 (HSF1) trimer. In the experiments [24], the time courses of HSF levels were obtained for three different temperatures, namely, 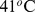, 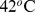, and 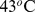, shown in red circles in (A), (B), and (C), respectively. For a very high temperature (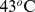), HSF levels reach a new steady state. Fitted time series of HSF13S levels (continuous line) are shown for three different 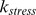 that correspond to the stress values of (A) 0.4136, (B) 0.9859, and (C) 1.2066. The percent conversion of the time series is calculated as 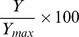, for all the dynamical variables, and Y is both experimental and simulated time series.

The continuous heat-shock experiments carried out by Abravaya et al. [24] on HeLa S3 cells resulted in the generation of an active HSF1 monomer followed by the rapid formation of a trimer that binds to HSE. At 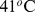, Abravaya et al. [24] observed relatively no changes in HSF1 levels, whereas at 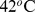, HSF1 increased rapidly, followed by a slow attenuation to reach a normal physiological concentration. At 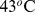, there was a rapid rise in HSF1 level, but the attenuation was completely absent so that it remained at a very high concentration (Fig. 2). The kinetic equations corresponding to the kinetic steps are given in Text S1.

#### Module 2: Phosphorylation-dephosphorylation reactions of the HSF13S:HSE complex

PdP reactions of the HS13S:HSE complex are an important step in the network construction that induces highly active transcription factors in response to stress signals. The serine and threonine residues in HSF1 have been implicated as plausible phosphorylated sites that enhance transcriptional response [32], but exactly how many stepwise phosphorylation reactions take place is not known. We assumed that the complex is twice phosphorylated, at both serine and threonine residues, by the kinase MK3 and dephosphorylated by the protein phosphatase-5A (PP5), a co-chaperone of the HSP molecular network [27]. Markevich et al. [26] have studied a constellation of dual PdP reactions that are either processive or distributive (see Burack et al. [33], and Ferrell et al. [34] for detailed explanations), and one such stepwise processive kinetic mechanism of dual PdP reactions was shown. These detailed PdP mechanisms can exhibit ultrasensitivity, hysteresis, and bistability for the choice of parameters. We adapted here two such constellations of dual PdP reactions for which the kinetic steps, and the corresponding equations are given in Text S2.

**Figure 3:**
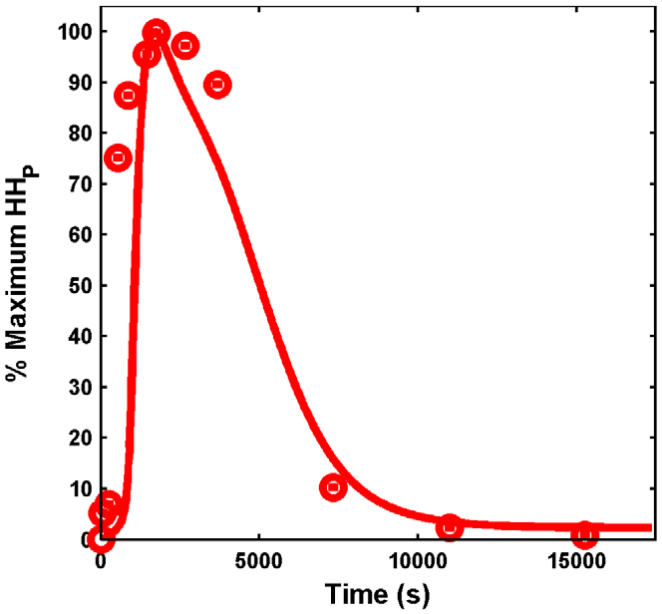
Fitted and experimental time series of the phosphorylated HH complex. Simulated mono-phosphorylated 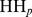 (shown in continuous line) for 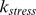 value of 0.9557, and the experimental data (shown in red circles) is taken from [25]. The percent conversion of the time series is calculated as 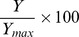, for all the dynamical variables, and Y is the simulated time series.

The experimental time course of the phosphorylation reactions indicates that there is a rapid rise in the phosphorylation of the complex followed by a very slow attenuation [25], but presently it is not clear whether the complex is mono- or multi-phosphorylated. Because the bifurcation analysis of the PdP reactions exhibited bistability, and the information regarding the number of PdP reactions from the experiments is not exactly known, we took the simulated time course of mono-phosphorylated complex (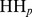) for fitting the experimental data (Fig. 3).

#### Module 3: Formation of the protein HSP90 and its conversion from the open to the closed conformation

HSP90 undergoes a stepwise conformational change either to bind to the client proteins or to activate the substrates involved in the reaction. HSP90 structure has three conserved domains; a C-domain, a middle or M-domain and, a N-domain. HSP90 forms a closed conformational dimer that results in the self-association of C, M, and N domains that are highly dynamic and flexible [35], [36]. C-C binding forms rapidly, but N-N binding is a slow step process and it is a subject of considerable study. Recently, Hessling et al. [37] captured ATP-assisted step-wise transformation from an open to a closed N-N lid conformation by Fluorescence Resonance Energy Transfer (FRET) experiments, and the time course data obtained were fitted to a model to extract the kinetic constants. Hessling et al. [37] also found from the experiments that two intermediate steps (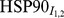 in the equations, Text S3) were involved in the formation of closed HSP90 conformation to attain a stable form that has a very high affinity for client proteins. In the present circuit, the client protein is the HSF1, which is sequestered by the closed conformation of the protein HSP90. HSP90 also requires the assistance of co-chaperone p23, which stabilizes the ATP-bound HSP90 to process the client protein, but this important step will be incorporated in the future along with the other important chaperones and co-chaperones.

**Figure 4:**
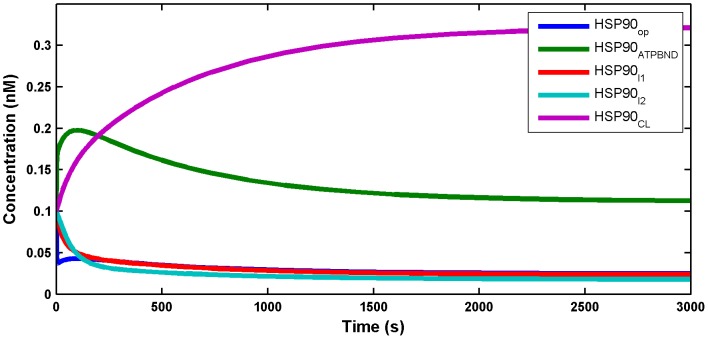
Time course of the various forms of HSP90. Simulated time course of the open, closed, and the intermediate forms of the HSP90 concentrations using the equations described in module 3. The initial concentration chosen for all the dynamical variables was 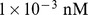 and they were allowed to evolve to the equilibrium point simulating a relaxation experiment.

The kinetic equations and the parameters that were used in Hessling et al. [37] to fit the FRET experimental data for the conversion of open to closed HSP90 conformation were retained for the present work. The kinetic and rate equations from Hessling et al. [37] are shown in Text S3, and the simulated time course is shown in Fig. 4.

### Parameter estimation

The kinetic parameters for modules 1 and 2 were unknown and we estimated them from the experimental data. The corresponding parameters for module 3 were taken from the literature [37]. For the sake of accuracy, the three modules were considered as a coupled system and the entire set of 27 ODEs was used to fit the 53 parameters of modules 1 and 2 to four time dependent experimental data series corresponding to HSF13S at three different temperatures (41, 42, and 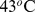) and to the mono-phosphorylated HHp complex at 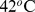. Temperature is the externally modulated stress parameter for the HSF13S data available in the literature [24] while in our model it corresponds to a different physiological and psychological source of stress. In order to be able to use this data, a different value for 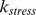 (representing the strength of the stress) was estimated for each of the temperatures expecting it to be higher for higher temperatures. Kline and Morimoto [25] studied the phosphorylation of HSF trimer-HSE complex in HeLa S3 cells at 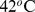. The type of cells and the experimental protocol for the phosphorylation study were the same those used in Abravaya's [24] study for determining HSF levels at three different temperatures. Since the protocols and cells used in both experiments are the same, we have simultaneously used the time series obtained from both experiments for fitting the parameters.

Parameter estimation, is a key step in the development of reliable dynamic models. Given a model structure and a set of experimental data, the objective of parameter estimation is to calibrate the model so as to reproduce the experimental results in the best possible way. The parameter estimation problem is stated as the optimization of a scalar cost function, 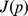, which measures the goodness of the fit with respect to the model parameters 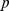. This function consists of a weighted distance measure between the experimental values and the predicted values for those variables. In this work, the cost function was defined as the least squares function resulting from the sum of the squared distances between the experimental and predicted values for HSF13S at each of the sampling points for the three different stress levels and the HHp at a single stress level:
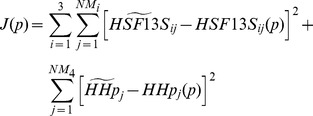
(14)where 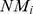 is the number of measures for experiment 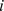 (i = 1,2, 3 for the HSF13S experiment at 41, 42, and 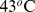, respectively, and i = 4 for the experiment measuring HHp), 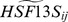 is the experimental value of the cortisol for the experiment 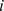 at the sampling point 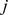, and 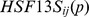 is the model prediction and analogously for HHp (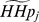 represents the experimental value and 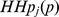 the model prediction). Since both HSP13S and HHp are found in the literature in arbitrary scales, the percentage with respect to the maximum value was considered for the fitting.

Due to the nonlinearities of the model equations, the resulting parameter estimation problem is multimodal; therefore, global optimization methods are required to avoid convergence to local solutions [38]. In this work we used the stochastic global solver SSm [39], which has been shown to be a powerful metaheuristic for parameter estimation in biological process. The parameter estimation as well as the sensitivity and the correlation analyses were performed with the help of SensSB [40], a Matlab®-based software toolbox for the development and sensitivity analysis of systems biology models.

Despite the use of a potent global optimization solver, fitting the parameters of such a large model is not an easy task. Moreover, due to the limited and noisy data available and the stochastic nature of SSm, different runs of the solver led to different sets of parameters with similar accuracy of the fitting. Nevertheless, the set of parameters obtained presenting the smallest cost function value was the one used for the later analysis and the one reported in Table 1. Fig. 2 represents the HSF13S model prediction versus the experimental data showing a fairly good agreement for two of the experimental conditions (42 and 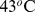), but for 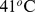, the fitting is poor. This is likely due to the fact that the data for 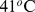 is highly noisy and variable in comparison to the other temperatures. As we simultaneously fitted the data for all the three temperatures for HSF1 as well as for the phosphorylation reactions, this is the best time series that could be obtained. The estimated values of 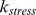 are 0.4136, 0.9859, and 1.2066, respectively, as expected since a higher temperature implies a higher level of stress. The model is able to qualitatively capture the dynamics as well as the new steady state achieved after exposure to a very high temperature (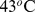). The trend of the HHp concentration was also properly fitted as shown in Fig. 3. Importantly, the 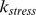 value estimated for fitting HHp at 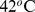 is 0.9557, which is very close to the value of 0.9859 for the data fitted for HSF1 at 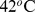, indicating the goodness of the fit.

**Table 1:** Kinetic parameters used in the bifurcation analysis and numerical integration.

Number	Constants	Values	Number	Constants	Values
1	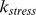 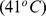	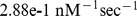	32	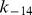	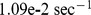
2	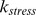 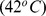	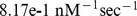	33	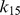	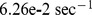
3	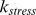 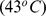	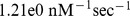	34	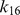	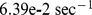
4	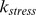 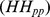	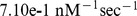	35	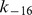	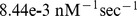
5	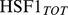	2.89e+2 nM	36	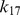	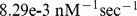
6	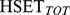	1.32e+3 nM	37	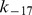	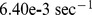
7	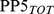	9.20e+1 nM	38	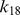	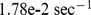
8	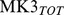	3.91e+1 nM	39	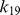	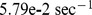
9	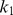	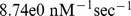	40	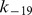	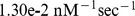
10	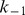	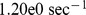	41	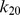	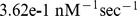
11	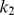	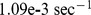	42	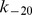	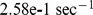
12	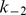	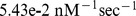	43	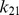	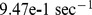
13	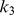	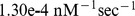	44	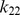	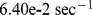
14	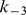	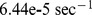	45	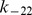	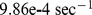
15	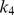	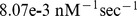	46	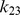	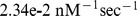
16	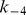	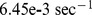	47	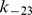	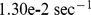
17	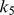	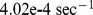	48	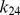	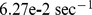
18	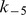	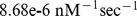	49	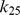	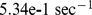
19	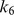	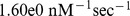	50	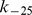	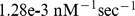
20	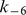	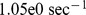	51	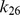	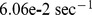
21	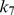	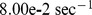	52	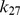	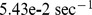
22	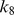	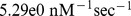	53	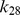	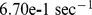
23	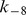	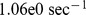	54	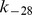	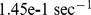
24	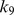	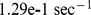	55	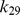	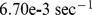
25	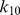	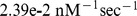	56	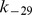	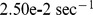
26	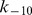	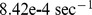	57	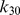	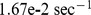
27	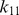	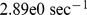	58	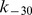	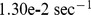
28	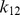	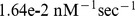	59	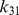	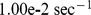
29	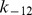	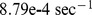	60	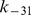	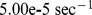
30	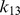	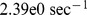	61	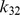	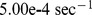
31	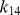	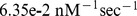	62	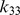	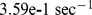

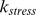
 is the bifurcation parameter used in the simulation of the full ordinary differential equation (ODE) model. All the parameter values estimated by global optimization as described in the Parameter estimation section, except 53–61, which are taken from [37].

**Figure 5:**
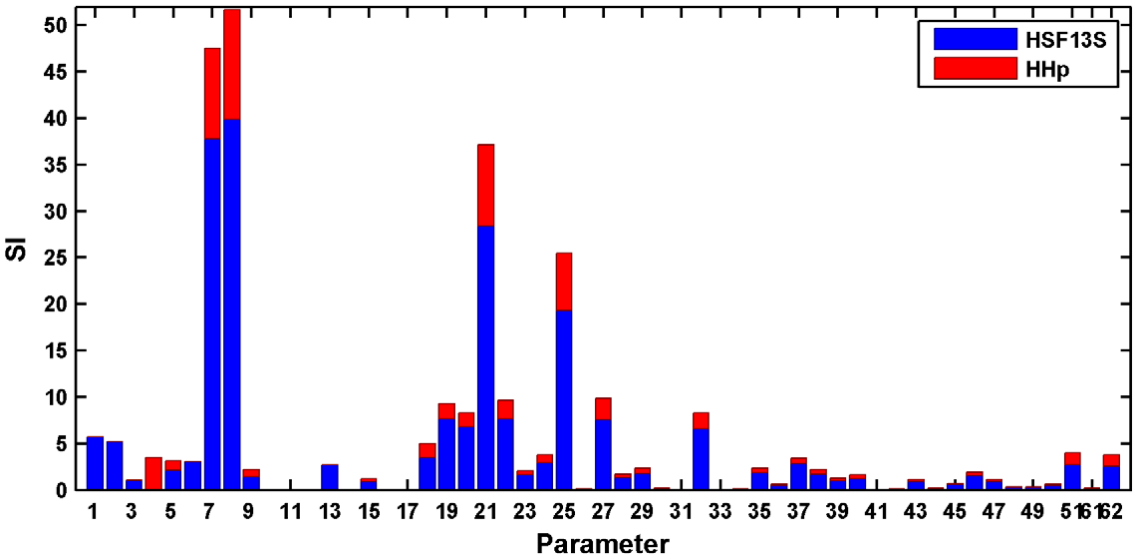
Local relative sensitivity analysis for the estimated parameters. The bars show in blue the sensitivity due to the effect of the parameters on HSF13S and in red the corresponding to HHp. As expected, there are several parameters having very little influence on the measured states, therefore, the parameter values obtained from the global optimization have to be taken with caution. Most of the parameters that appear insensitive in this analysis are sensitive to other species, thus experimental data of these intermediate states would help to better estimate these values. The numbers on the abscissa corresponds to the kinetic constants given in Table 1.

In order to gain further insight into the dynamics predicted by the model, a local sensitivity analysis was performed for the optimal set of parameters represented in Table 1. Parametric sensitivity analysis aims to investigate how a change in the parameters affects the model output. In this case, we are interested on analyzing this influence on the measured states, namely HSF13S and HHp. In this study we used relative sensitivity indices, computed by multiplying the partial derivative (the absolute sensitivity function) by the nominal value of the input and dividing it by the output value. The relative sensitivity index (SI) of the model outputs to variations in the parameter 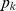 evaluated for the optimal set of parameters 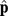 is given by:
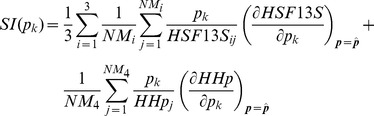
(15)
Fig. 5 shows in blue the sensitivity due to the effect of the parameters on HSF13S and in red the corresponding to HHp. As expected, there are several parameters (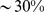) having very little influence on the measured states, therefore, the parameter values obtained from the global optimization have to be taken with caution. Most of these parameters that appear insensitive in this analysis are sensitive to other species, thus experimental data of these intermediate states would help to better estimate these values. For example, the reaction rate of the backward reactions of the PdP have a small influence in the measured states but more information about the intermediate states should be needed in order to asses the importance of these steps.

**Figure 6:**
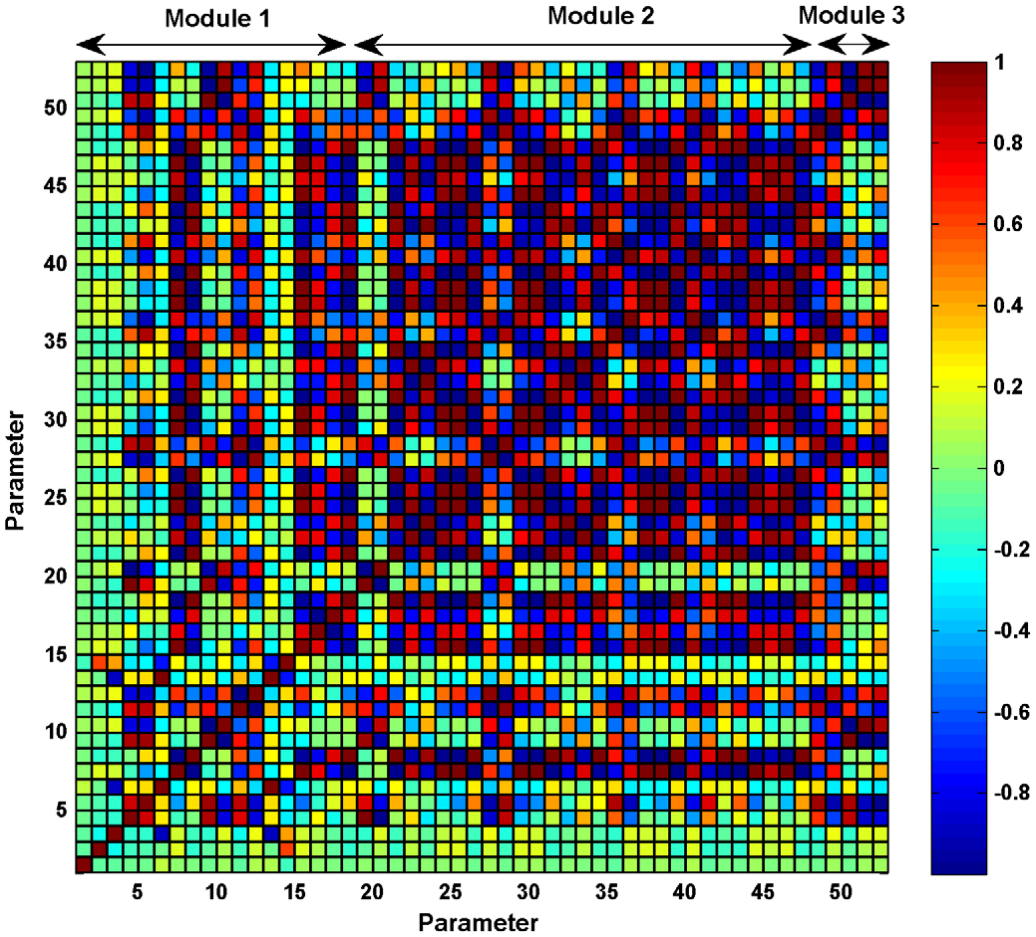
Correlation matrix for the 53 estimated parameters. The correlation matrix shows a strong positive correlation (+1) or a strong negative correlation (−1) between several sets of parameters indicating that the effect of one parameter can be compensated by a change in the strongly correlated parameter. This points to the same direction as that of the sensitivity analysis, emphasizing the need for more experimental data of intermediate species to uniquely identify the values of the parameters.

Moreover, the a posteriori identifiability or estimability of the parameters has been studied by computing the correlation between the dynamic sensitivities of the measured states at the experimental sampling points as described in [41], [42]. The correlation matrix (see Fig. 6) shows a strong positive correlation between several sets of parameters. These strong correlations, together with the low sensitivities found for some of the parameters, indicate that more experimental data of intermediate species would be needed to uniquely identify the values of the parameters. Moreover, we can see important correlations between the parameters of module 1 (from 1–10) and the parameters of module 2 (11–53), emphasizing the need for a joint estimation of the whole set of parameters.

It is also interesting to note that, due to the identifiability problems and the noise in the data and despite using a global solver, different values for some of the parameters are obtained for each run. As a consequence of this we expect the dynamics also to be affected and this is studied through bifurcation analysis in the following section.

### Bifurcation analysis of the full HSP90 network with a negative feedback loop: mushroom dynamics

**Figure 7:**
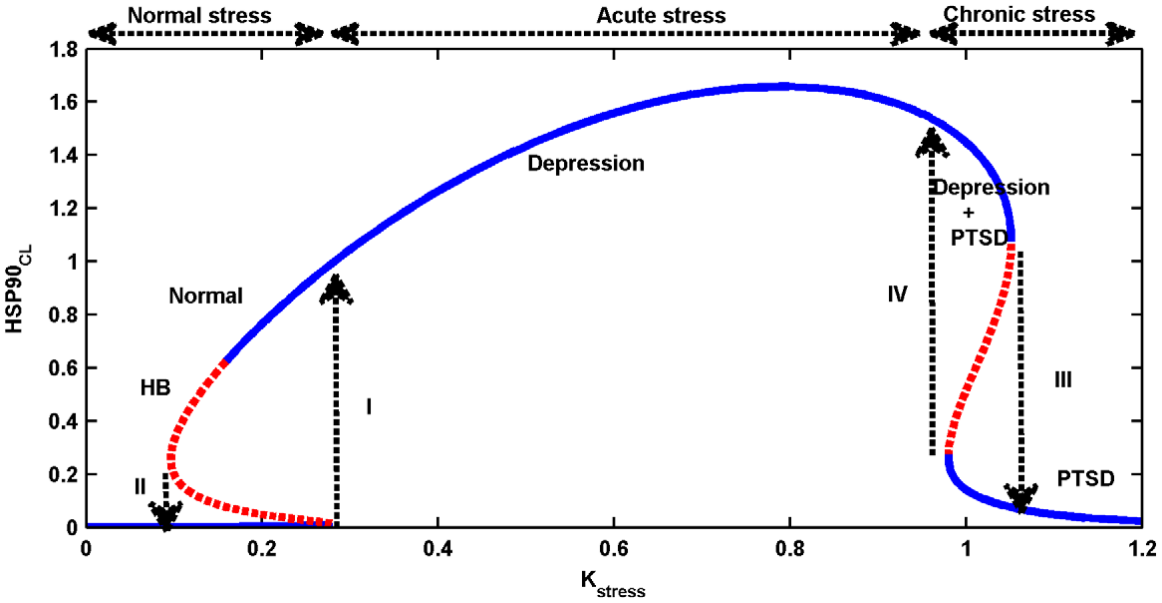
Occurrence of mushroom dynamics in the full ordinary differential equation (ODE) model with 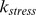
 **as the bifurcation parameter.** Two bistable forms, S-shaped (on the left, normal stress), and Z-shaped (on the right, chronic stress) are present together, giving rise to a mushroom when the bifurcation parameter 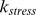 is varied. During normal stress, 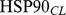 increases and crosses the threshold I, to transit to another stable steady state with a high concentration. When the stress level decreases, 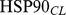 concentration also decreases gradually from a high to a low concentration level by crossing the threshold II, and retaining the normal homeostatic conditions. This happens in the S-shaped bistable form. In contrast, during acute stress, 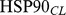 crosses the threshold I and correspondingly it increases; this is attributed to depression. Further increase in acute stress results in the moderate increase of 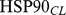, wherein post-traumatic stress disorder (PTSD) develops along with depression. During chronic stress, 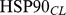 abruptly drops to a very low level by crossing the threshold III. This is attributed to PTSD, where high stress level results in a very low concentration of 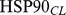. This event occurs in the Z-shaped bistable part of the mushroom dynamics. If the stress level decreases from a very high value, i.e, 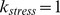, 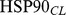 slowly increases from a very low concentration, but once it crosses the threshold IV, there is an abrupt jump in the concentration. The stable steady state is shown as a continuous blue line, whereas the unstable steady state is shown in dashed red lines.

We performed a bifurcation analysis of the full ODE model shown in Text S4, with 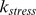 as the bifurcation parameter. To describe the dynamics that arise due to the effect of stress on the HSP90 network, we chose 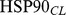 as the dynamical variable. Mushroom, wherein the S-shaped and Z-shaped forms of the bistable dynamics are present together, was observed (Fig. 7). Mushroom dynamics were extensively studied in many chemical reactions under continuous-flow stirred-tank reactor (CFSTR) conditions [43], [44], and other researchers applied singularity theory to determine the presence of mushrooms and isolas in nonlinear ODE models [45]. In neuro-biological systems, Song et al. [46] applied singularity theory to the ODE model of the serotonin network to explain the occurrence of long-term memory in the mollusk *Aplysia* system, and observed a wide variety of bistable dynamics that include hysteresis, irreversible transitions, mushrooms, and isolas.

We identified three different regions in the bifurcation diagram based on stress intensity, and related the three regions to normal, acute and chronic stress. Under a normal stress level, 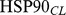 increases from a very low to a high concentration by crossing the threshold I (Fig. 7), while during de-stress, 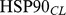 decreases, and returns to the normal level by crossing the threshold level II. This sudden jump and fall of 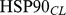 levels occur in the S-shaped part of the mushroom bifurcation. At an extremely high level of stress, 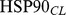 crosses the threshold III, and drops to a very low concentration. High stress with a low level of 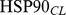 indicates a complete breakdown of the network homeostasis. These bistable dynamics occur in the Z-shaped part of the mushroom dynamics. We attribute this high stress level to “chronic stress”. For an intermediate level of stress, high concentration of 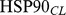 is attained through two different routes: (i) by crossing the threshold I in the S-shaped form when the stress is increased from a very low to a high intensity, or (ii) by crossing the threshold IV in the Z-shaped form when the stress level changes from a very high to a low intensity. We attribute this intermediate stress level to “acute stress”.

**Figure 8:**
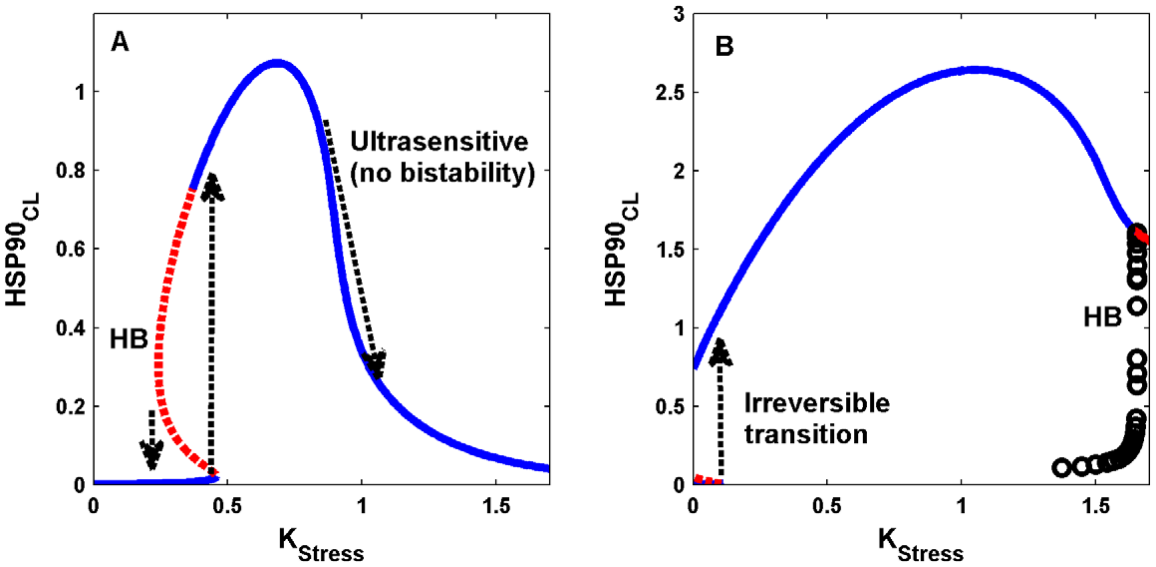
Observation of different dynamical scenario for the parameter set obtained from fitting the time series data by global optimization. Loss of mushroom-like dynamics in the full ordinary differential equation (ODE) model with 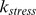 as the bifurcation parameter. (A) The Z-shaped bistability is lost, but ultrasensitivity is retained. A mushroom-like dynamics is possible without any memory effect at high chronic stress due to loss of Z-shaped bistability. (B) Irreversible transition in the S-shaped bistability is observed. Z-shaped bistability is lost and it is replaced by Hopf bifurcation with a very low period. The parameters used to simulate the dynamics for each of the cases are given in a separate file (Model file S2).

In Fig. 8, we also show two other possible dynamical scenarios among various possibilities for two different parameter sets obtained from fitting the time series data by global optimization. It can be seen that the Z-shaped bistability is lost, but replaced by ultrasensitive stable steady state for one choice of the parameter set. In another case, an irreversible transition is observed in the S-shaped bistability, and the Z-shaped bistability is replaced by a Hopf bifurcation. This is biologically implausible as in many other cases where negative steady state concentration was observed for other dynamical variables, and we have discarded those scenarios. To summarize, we have not completely explored all the dynamical scenarios and its implications under acute and chronic stress, and this is relegated to future work. For the present case, only mushroom dynamics are considered.

### Comparison with the earlier models of heat shock network

It is pertinent to discuss here briefly the earlier models [7]–[10] of heat shock network to compare them with the present model. All the earlier models include three modules: (i) the HSF trimerization reactions, (ii) the complexation of the trimer with HSE, and (iii) the production of HSP from mRNA. However, the details in framing the network and the corresponding mathematical models are different. Mostly, the purpose of these models was to account for the double heat shock response of the system obtained from the experiments, and make predictions from the model. Rieger et al. [8], developed a minimal model of HSP70 network to understand its dynamics under stress, to evaluate the role of critical steps in the network that affects the expression of molecular chaperones, and to evaluate the predictive abilities of the model. However, they have not addressed the role of the trimerization reactions, multiple PdP reactions, the timing of trancriptional/translational machinery etc., but only indicated that their model offered a framework to introduce all details. Their model was either based on the laws of mass action kinetics or the variant of Michaelis-Menten equation, namely the Goldbeter-Koshland function, and the kinetic parameters were determined based on the experimental observations.

The present model is detailed and differs from the earlier models in many ways. All the three modules are considered, but are elaborately constructed and analyzed in detail. The trimerization reactions are assumed to take place in a stepwise fashion. Detailed PdP reactions of the trimer-HSE complex are considered, and are shown to give rise to bistability. This is a new and important step in comparison to the earlier models. The PdP reactions are built based on the work of Kholodenko et al. [26], and though the kinetic steps and equations are complex in nature, this is taken to capture the rapid phosphorylation-dephosphorylation that takes place in the serine and threonine residues in the HH complex. The hidden positive feedback loop in the PdP reactions plays a vital role in decision making processes in our model. The kinetic parameters of the modules 1 and 2 are estimated through global optimization. The model fits the data of HSF1 trimer at three different temperatures well, and this is due to the occurrence of a saddle-node bifurcation in the model. Therefore, one of the predictions from our model is that at temperatures 41 and 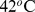, the system is present in the lower stable steady state, while at 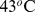, the system transits to the upper stable steady state. The model fits the phosphorylated HH complex well. However, we fit the experimental data to simulated mono-phosphorylation, since we do not know from the experimental time series whether HH complex is mono or multi-phosphorylated. However, the experiments also point out that HH complex is multi-phosphorylated for which experimental time series are not available. This is the classic case of identifiability problem when fitting the data, and as a consequence, most of the parameters of PdP reactions appear to be insensitive. Therefore, with the present data, minimal model of Rieger et al. [8] or any other model may be sufficient to explain the dynamics of the network. However, our detailed model fits the data well in comparison to other models. The post translational modification of HSP90, that involves changing the conformation from open to closed form through intermediate steps is considered, and the kinetic parameters used in modeling this module are based on FRET experiments. The new aspect from the dynamics point of view is that, it is shown for the first time that the model can exhibit exotic complex dynamics, namely a mushroom, where S and Z shaped bistable dynamics are present together. Importantly, mushroom dynamics are mapped onto normal and pathological systems, and speculative molecular mechanisms (see section 2.5) are advanced to explain the importance of negative feedback loop in the HSP90 network. This is the novel aspect of the model that is used to make sensible predictions about various neuro-psychiatric disorders, which has hitherto not been considered.

### Speculative molecular mechanisms on the occurrence of mushroom dynamics

**Figure 9:**
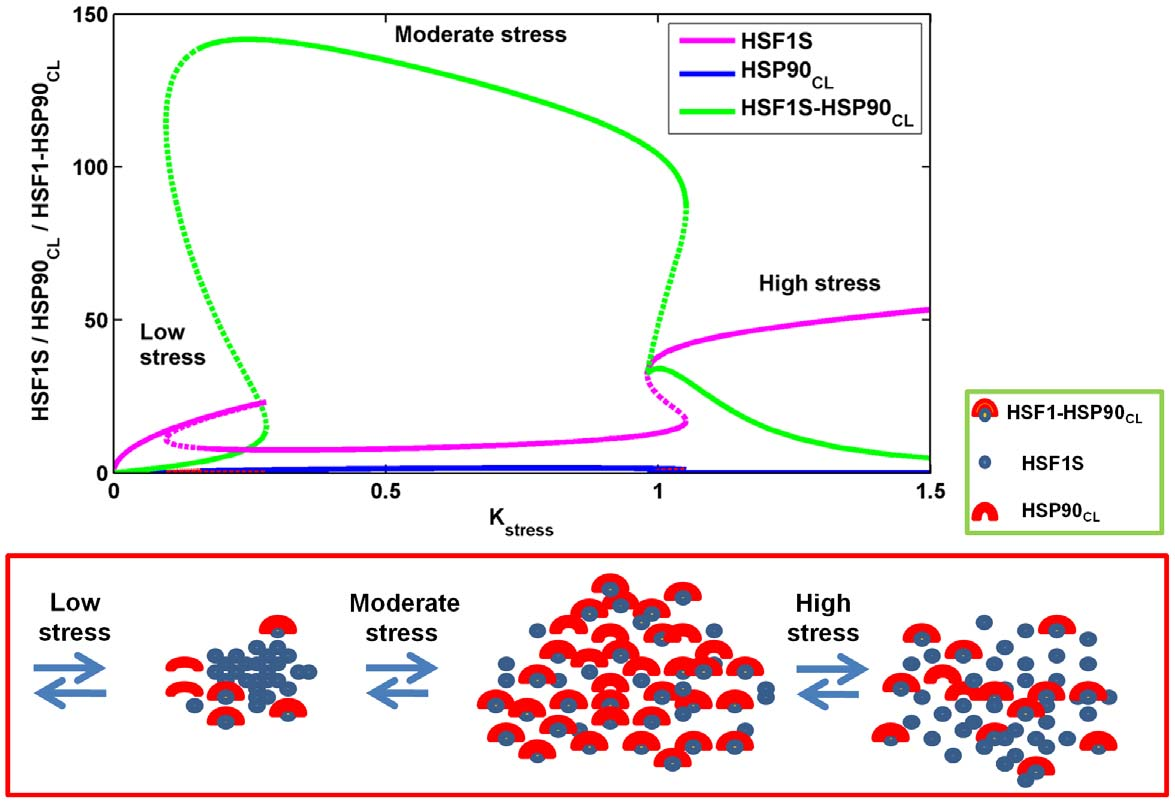
Dynamics of stress-initiated molecular events in heat-shock protein 90 (HSP90) production. Three different levels of stress, low, moderate, and high, are considered in mapping the molecular events that drives the mushroom dynamics of the HSP90 network using the bifurcation diagram. An illustration of the molecular events corresponding to the occurrence of mushroom dynamics in terms of heat-shock factor 1 monomer (HSF1S, blue filled circle), the closed conformation of heat-shock protein 90 (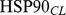, in yellow-filled red semicircle) and 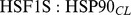 complex (in yellow-filled red semi-circle bound to blue filled circle) are shown in the lower panel. A low level of stress increases the inactive HSF1, and converts it rapidly into an active form, HSF1S. 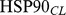 is present at a low level due to the time taken for its production. An increase in stress to a moderate level results in further production of active HSF1S. This in turn oligomerizes, and through a sequence of PdP reactions, produces more 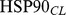. This negatively regulates its own production by rapidly forming a complex with the inactive HSF1 monomer, and results in a drastic reduction of its own concentration. For a very high stress level, 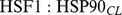 complex breaks down rapidly, and the inactive HSF1 is again converted into a more active form, HSF1S.

**Figure 10:**
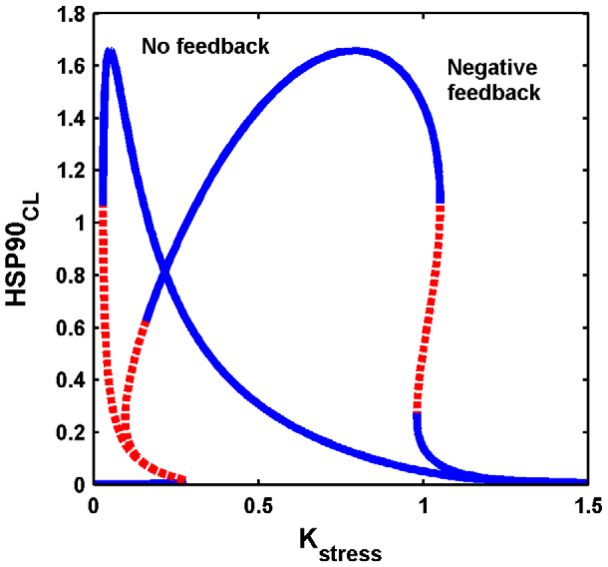
Bifurcation diagram in the absence of the negative feedback loop. One-parameter bifurcation diagram is constructed in the absence of negative feedback loop by silencing the kinetic constants 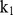 and and 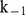 to zero. Only the S-shaped part of the mushroom is seen, and the Z-shaped part is absent. This indicates that to generate Z-shaped bistable part, a strong negative feedback loop is important, and this strong feedback loop may be responsible for the occurrence of depression and post-traumatic stress disorder (PTSD) (see also Fig. 7).

We propose a molecular mechanism to explain the molecular events that give rise to S-shaped and Z-shaped bistable parts of the mushroom dynamics when stress intensity is varied. We describe this using the bifurcation diagram shown in Fig. 9 by taking three important dynamical variables into consideration, namely 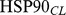, HSF1S, and the 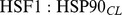 complex which form a core negative feedback loop in the network. 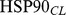 and the 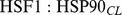 complex form a mushroom, whereas HSF1S forms an inverted mushroom. Again, we studied the dynamics for three different stress intensities: low, moderate, and high. In the absence of any stress, HSF1 sequesters 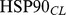 to form the complex 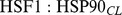, and therefore the active form of HSF1, namely HSF1S, cannot initiate any reaction in the network. All three species, 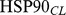, HSF1S, and 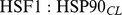, remain at very low concentrations. At a low level of stress, HSF1S increases, because stress breaks down the 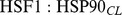 complex, and converts the inactive oligomer HSF1 into the active form, HSF1S. As oligomeric HSF1S increases rapidly, mRNA90 and 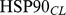 also increase, but with a delay due to the time taken for the PdP reactions, transcriptional-translational processes, and the conversion of an open to a closed conformation of 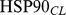. This occurs in the S-shaped bistable part of the mushroom dynamics, and the bistability is due to the PdP reactions of the trimer. At intermediate, or “acute” stress levels, 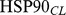 sequesters the inactive HSF1 to form more of the complex 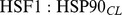, which reaches a very high concentration. This event occurs between S-shaped and Z-shaped parts of the mushroom dynamics. At a high or “chronic” stress level, more 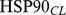 is produced, and the negative feedback loop, due to the formation of the complex between 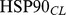 and HSF1, becomes stronger. Simultaneously, the 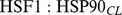 complex breaks down rapidly due to high stress, which results in the generation of more HSF1S. This event occurs at the Z-shaped part of the mushroom. We speculate that the silencing of the negative feedback loop in the HSP90 network leads to a selective loss of the Z-shaped bistable part of the mushroom dynamics that may result in a very low concentration of 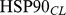, whereas the S-shaped part of the mushroom dynamics remains intact. To support this speculation, we constructed a bifurcation diagram in the absence of the negative feedback loop; as predicted, the Z-shaped part of the mushroom dynamics was absent, and the concentration of 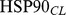 dropped down to a very low value (Fig. 10) for a very high level of stress. This result clearly shows that the strong negative feedback loop formed due to sequestration of 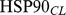 with inactive HSF1 is responsible for the occurrence of the Z-shaped bistable part of the mushroom dynamics.

### Acute and chronic stress, depression, and post-traumatic stress disorder: a dynamical system hypothesis on the role of HSP90

In animal models, acute and chronic stress induces different behaviorial, physiological, and molecular expressions in comparison to normal levels of stress, and importantly, it has been shown that gene expression under fear conditioning was strongly dependent on the brain regions, namely hippocampus, amygdala, and the pre-frontal cortex (PFC) [47]. The genomic responses in different brain regions were also shown to be strongly dependent on the type of fear conditioning protocols employed. Recently, Zhang et al. [48] performed inescapable tail shock experiments that suggested that stress increases the HSP90 concentration in the PFC area of rat brains more than in that of unstressed rats, but the exact mechanism has not been elucidated. Sirri et al. [13]. carried out the gene expression profiling of the C57Bl/6N mice hippocampus after performing a trace fear conditioning experiment, and found that many of the protein folding and protein quality control transcription factors were elevated. In humans, based on SNP experiments, FKBP5, the co-chaperone of HSP90 is strongly implicated in PTSD.

Based on the results of the fear conditioning experiments, a plausible explanation on the role of HSP90 in fear conditioning can be provided. Fear conditioning induces fear memory through the promotion of synaptic plasticity. For synaptic plasticity, the delivery of neurotransmitters through the receptors from pre-to-post synaptic terminals is absolutely necessary for long-term memory of fear in different brain regions for efficient communication among various neurons. HSP90 is an abundant and constitutively expressed molecular chaperone in neurons [49] along with other complementary co-chaperones like HSP70 in the rat brain. Evidences from experiments indicate that the HSP90 is a classical molecular chaperone necessary for efficient neurotransmitter release at the pre-synaptic terminal, and is known to catalyze and promote the assembling and dissembling processes required for AMPA receptor recycling [50]. AMPAR itself is known to play a strong role in synaptic plasticity under fear conditioning [51]. Therefore, HSP90 is necessary for efficient recycling of receptors in the hippocampus and amygdala for strong fear memory. Another compelling evidence is that the co-chaperone FKBP5 that is strongly correlated with PTSD, along with HSP90 helps GR bound cortisol in the HPA axis to translocate to the nucleus for further downstream regulation [21] for autonomic responses. Consolidating these evidences, HSP90 plays a strong role in fear memory by not only translocating receptors from pre to post synaptic terminals in the hippocampus or the amygdala, but also chaperoning the glucocorticoid receptor bound cortisol to facilitate the fear learning process, which when unregulated under stress results in pathological conditions like depression.

Based on these observations from gene expression profiling, we hypothesize that the mushroom dynamics obtained from the bifurcation analysis of HSP90 network can be used to predict the occurrence of both depression and PTSD that may be true for both the animal and human models. Specifically, we hypothesize that the S-shaped bistable dynamics in the mushroom can be related to the normal system while the Z-shaped dynamics can be related to the occurrence of both depression and PTSD in humans. Specifically, for the animal model, we relate three regions of stress, namely normal, acute, and chronic stress and its corresponding HSP90 concentrations, which are high, higher and low respectively in the bifurcation diagram. In the human model, we identify the same three regions of stress of the animal model, but the concentration of HSP90 is related to normalcy, depression and PTSD. To elaborate on this in terms of the HSP90 network, stress increases 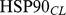 but de-stress decreases 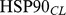 slowly to a very low concentration, as captured by the S-shaped bistable dynamics. However, if stress recurs frequently but at a moderate level, 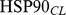 remains at a high concentration and lies between the S-shaped and Z-shaped parts of the bistable dynamics, which we predict is the region of transition from a normal condition to a comorbid disorder such as depression. This is the region of acute stress. This pathological condition can be reversed to a normal condition when the stress levels are reduced. In the presence of an extremely high and constant stress level, PTSD may also begin to develop along with depression. In such a case, there is a complete breakdown of homeostasis due to the strong action of the negative feedback loop in the HSP90 network that fails to release the active HSF1S to initiate the reactions to counteract the strong stress. This pathological event is related to the Z-shaped part of the mushroom dynamics, wherein there is an abrupt drop in the 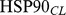 at very high stress levels, and the network fails to respond to any changes of the stress. We relate this scenario to the chronic stress. It remains to be confirmed whether, as predicted by this bifurcation analysis, the application of high-intensity stress in mammalian systems over a prolonged period results in HSP90 reaching a very low concentration.

## Discussion

The understanding of stress-induced molecular events in fear conditioned animals and its relation to various types of psychiatric disorders such as depression and PTSD in humans is still in its infancy. Systems-level understanding may provide better insight into the cause of these disorders that may lead to a precise diagnosis and intervention of disease. One network that alters significantly in any psychiatric disorder is the HSP90-chaperone network; the co-chaperones FKBP51 and FKBP52, changed significantly in patients with PTSD who were treated for childhood abuse [52]. There are few dynamical molecular models constructed to understand these disorders.

In our work, a small, yet detailed molecular network of HSP90, and the corresponding mathematical model were formulated not only to study the dynamics that occur due to acute and chronic stress in animal models, but also to relate them to psychiatric disorders such as PTSD, and its comorbid disorders such as depression. Importantly, we took into consideration the detailed phosphorylation-dephosphorylation reactions of the HSF13S:HSE complex that play a significant role in the generation of bistable dynamics. Oscillatory dynamics were also observed due to the presence of a negative feedback loop, but this was not probed, and was instead relegated to future work. The significance of the present dynamical analysis is the occurrence of a type of bistable dynamics, mushroom dynamics. A wide variety of bistable dynamics have been observed in many bio-chemical systems [53], and the mushroom is a type of bistable systems where both the S-shaped and Z-shaped hysteretic forms are present together. Here, we specifically interpret the presence of mushroom dynamics in the context of normal function and psychiatric disorders; i.e. the S-shaped hysteretic form to the normal conditions, and the Z-shaped hysteretic form to acute and chronic stress, and extended this model to psychiatric disorder such as depression and PTSD in humans. This is entirely different from the usual interpretation of bistable dynamics in the contest of psychiatric disorder; for example, Gupta et al. [54], in their bistable model for chronic fatigue syndrome due to stress, related one stable steady state to the normal conditions, and the other stable steady state to pathological conditions. Therefore, mushroom dynamics are a much more convenient way to capture the variations of HSP90 under three different conditions, namely, normal, acute and chronic stress.

A significant prediction made from our model is that, for a very high “chronic” level of stress, the drop of the 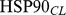 concentration to a very low level was attributed to PTSD in humans. We showed that this occurs in the Z-shaped bistable part of the mushroom due to the strong negative feedback loop in the network. In the fear tracing studies, elevated level of heat shock protein was observed immediately after exposure, but a reduction in concentration occurred after a considerable delay, and this was interpreted due to degradation. However, we can interpret the reduction in concentration due to two other possible mechanisms; fear extinction, or due to some feedback mechanisms operating in the network that reduce the concentration. For example, in the HPA axis, Yehuda et al. [55], [56] observed that the cortisol concentration drops to a low level between late night and early morning in persons with PTSD in comparison with normal or depressed persons [14]. This difference in the cortisol levels was predicted due to an enhanced negative feedback loop generated by cortisol in the HPA axis [57], and this was recently captured by mathematical modeling [58]. But cortisol, its corresponding type-I and type-II receptors (mineralocorticoid receptors and glucocorticoid receptors, respectively), and the co-chaperone FKBP5 are themselves regulated by the protein HSP90. Therefore, we studied here, as a first step, the dynamics of the HSP90 network and predicted a very low concentration of HSP90 for a very high stress level and related it to persons with PTSD. At present, there are no experimental data to account for our predictions, but the hypothesis is easily testable.

**Figure 11:**
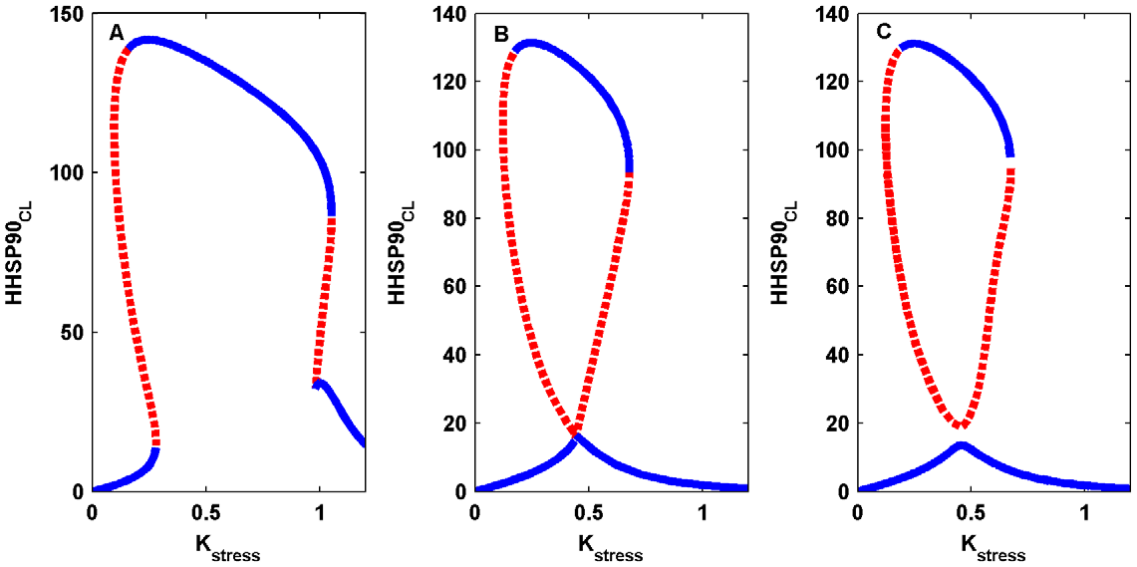
Transition from mushroom dynamics to an isola due to the variation in the phosphorylation-dephosphorylation (PdP) kinetic parameter. (A) Slowing down of the phosphorylation reaction is modulated by varying the kinetic constant 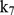 from 0.08 to 0.038, which results in the S-shaped, and Z-shaped forms coming closer to each other. (B) Further decrease of 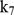 (0.0383) results in the mushroom touching the lower steady state and the lower limit points of S-shaped and Z-shaped forms of bistablity are merged together. (C) Isolated stable steady state along with unstable steady state is formed when the mushroom is pinched off from the lower stable steady state (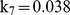). This is termed an “Isola”. The blue color denotes the stable steady state, whereas the unstable steady state is denoted by the red dotted lines.

It is well documented that PdP reactions are capable of exhibiting multistability [59]–[61] and chaos [62]. In the present work, the PdP-reaction network plays an important role in generating the S-shaped bistable part of the mushroom dynamics. With no PdP reactions in the network, mushroom dynamics are not possible because this is the only reaction that has an implicit positive feedback loop. Positive feedback loops are a necessary and sufficient condition to generate bistable dynamics for the choice of parameters [63], [64]. Sensitivity analysis also indicated that the kinetic parameters in the PdP reactions are highly sensitive, and therefore, we analyzed the effect of PdP kinetic parameters on the mushroom kinetics. When the rates of the phosphorylation reactions are changed in the model, the mushroom dynamics are also changed. This is shown by varying the kinetic constant 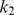, the rate of mono-phosphorylation reaction, which causes the dynamics to change from the mushroom to an isola (Fig. 11). To elaborate, as 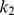 is reduced, the lower limit points in the S-shaped and Z-shaped parts of the mushroom come closer together (Fig. 11A), and when 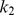 is reduced further, both the lower-limit points merge and touch the lower branch of the stable steady state solution (Fig. 11B). Further, in this sequence, when 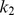 is reduced, we observe an isola in which two stable steady states are isolated from each other (Fig. 11C). If the system is in the lower stable steady state of the isola, the variation in the stress intensity will not have any impact on the network because 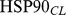 concentration level will always remain at a very low level. This is a pathological event that arises due to the variations in the production of phosphorylated complex. At present, there are no experimental data to account for this aspect of our model predictions.

To conclude, chaperones and co-chaperones play a vital role in many psychiatric disorders. Elucidating the pathway mechanisms along with understanding of the network dynamics can help to predict the variations of particular species in the network under acute and chronic stress in animal models, as well as distinguish comorbid disorders such as depression and PTSD. Depending on the stress intensity and the concentrations of HSP90 and HSF1S monomeric or oligomeric proteins, depression and PTSD can easily be distinguished. The protein HSP90 may also serve as a good candidate bio-marker for PTSD, but presently, the problem lies in the detection of this protein due to its differential expression in various subregions of the brain such as hippocampus and prefrontal cortex. In future work, the inclusion of co-chaperones like FKBP51 and FKBP52 along with the glucocorticoid receptors and cortisol in the network will provide further insight about the etiology of these disorders at the systems level.

## Supporting Information

Text S1
**Module 1: Kinetic steps and equations for the formation of the trimer and the negative feedback with** 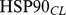 **.**
(PDF)

Text S2 **Module 2: Kinetic equations for the phosphorylation-dephosphorylation reactions of the HSF1 trimer-HSE complex (HH).**
(PDF)

Text S3 **Module 3: Production of the open form of HSP90 from mRNA90 and kinetic equations for the ATP-assisted conversion of HSP90 from an open to a closed conformation.**
(PDF)

Text S4 **Simulation of the full ordinary differential equation model.**
(PDF)

Model file S1 **Ordinary differential equation (ODE) file of XPPAUT used to simulate the bifurcation diagrams.**
(ODE)

Model file S2 **Parameters used to simulate the dynamics for each of the cases depicted in** **Figure 8** **.**
(XLSX)
