# Supplementary material for: A Detailed Modular Analysis of Heat-Shock Protein Dynamics under Acute and Chronic Stress and Its Implication in Anxiety Disorders
Source: PLoS One. 2012 Aug 22;7(8):e42958. doi: 10.1371/journal.pone.0042958 (PMC3425570; doi:10.1371/journal.pone.0042958)
Supplement: Text S1 — Module 1: Kinetic steps and equations for the formation of the trimer and the negative feedback with . (PDF) [file pone.0042958.s001.pdf]

Text S1

Module 1: Kinetic steps and equations for the formation of the trimer and the negative feedback with HSP90$_{CL}$.

Module 1 consists of the formation of active monomer, dimer, trimer and the sequestration of trimer with the closed form of HSP90. The active HSF1S monomer from the inactive HSF1 is formed only under intense stress that releases inactive HSF1 from the complex HSP90$_{CL}$. Complexation of HSF1 by HSP90$_{CL}$ results in a negative feedback by which HSP90$_{CL}$ regulates its own production. The kinetic steps and the equations for the negative feedback loop, and the release of active heat shock transcription (HSF1S) in the presence of strong stress are given as follows:

$$HSF1 + HSP90_{CL} \underset{k_{-1}}{\overset{k_1}{\longleftrightarrow}} HHSP90_{CL} \quad (1)$$

$$HHSP90_{CL} \underset{k_{-2}}{\overset{k_2}{\longleftrightarrow}} HSF1S + HSP90_{CL} \quad (2)$$

$$R1 = k_1[HSF1][HSP90_{CL}] - k_{-1}[HHSP90_{CL}] \quad (3)$$

$$R2 = k_2[HHSP90_{CL}] - k_{-2}[HSF1S][HSP90_{CL}] \quad (4)$$

The active HSF1S monomer is converted into a dimer and a trimer in a stepwise fashion. Proctor et al. [1], in their model, assumed that the formation of HSF1S dimer was a slow forward rate process, while its dissociation to monomer was a fast reversible process. We have also made a similar assumption, but for the sequestration reaction and not for the dimerization reactions; i.e., we assumed a strong sequestration of HSF1S by HSP90$_{CL}$ ($k_{l21} >> k_{l2}$) because of the strong affinity of HSF1S to HSP90$_{CL}$. Therefore, only a very high level of stress can dissociate HSF1S from HSP90 to form a dimer slowly, followed by a rapid binding of HSF1S monomer to its dimer that leads to the formation of the trimer. The kinetic equations for the formation of the trimer, and the complexation of the trimer with the HSE promoter, denoted by HH, that occurs in a stepwise fashion are given below:

$$2HSF1S \underset{k_{-3}}{\overset{k_3}{\longleftrightarrow}} HSF12S \quad (5)$$

$$HSF12S + HSF1S \underset{k_{-4}}{\overset{k_4}{\longleftrightarrow}} HSF13S \quad (6)$$

$$HSF13S + HSE \underset{k_{-5}}{\overset{k_5}{\longleftrightarrow}} HH \quad (7)$$

$$R3 = 2k_3[HSF1S]^2 - k_{-3}[HSF12S] \quad (8)$$

$$R4 = k_4[HSF12S][HSF1S] - k_{-4}[HSF13S] \quad (9)$$

$$R5 = k_5[HSF13S][HSE] - k_{-5}[HH] \quad (10)$$

HSF1 is the inactive heat-shock factor, HSF1S, HSF12S, and HSF13S are the active heat-shock factor monomer, dimer, and trimer; HSE is the heat-shock element promoter; HH is the complex HSF13S:HSE; and ϕ is the degraded product.

Rate equations for the simulation of Module 1

$$\frac{d[HSF1S]}{dt} = k_{stress} + R2 - R3 - R4 \quad (11)$$

$$\frac{d[HSF12S]}{dt} = R3 - R4 \quad (12)$$

$$\frac{d[HSF13S]}{dt} = R4 - R5 \quad (13)$$

k_{stress} is the bifurcation parameter.

References

1. Proctor CJ, Sőti C, Boys RJ, Gillespie CS, Shanley DP, et al. (2005) Modelling the actions of chaperones and their role in ageing. Mech Ageing Dev 126: 119-131.
