# Supplementary material for: A Detailed Modular Analysis of Heat-Shock Protein Dynamics under Acute and Chronic Stress and Its Implication in Anxiety Disorders
Source: PLoS One. 2012 Aug 22;7(8):e42958. doi: 10.1371/journal.pone.0042958 (PMC3425570; doi:10.1371/journal.pone.0042958)
Supplement: Text S2 — Module 2: Kinetic equations for the phosphorylation-dephosphorylation reactions of the HSF1 trimer-HSE complex (HH). (PDF) [file pone.0042958.s002.pdf]

Text S2

Module 2: Kinetic equations for the phosphorylation-dephosphorylation reactions of the HSF1 trimer-HSE complex (HH)

We consider the stepwise dual phosphorylation-dephosphorylation reactions of the complex HH as module 2. The kinetic equations of the phosphorylation-dephosphorylation reactions of the complex HH by the kinase and phosphatase are given as follows:

$$HH + MK3 \underset{k_{-6}}{\overset{k_6}{\longleftrightarrow}} HHMK3 \tag{1}$$

$$HHMK3 \xrightarrow{k_7} HHp + MK3 \tag{2}$$

$$HHp + MK3 \underset{k_{-8}}{\overset{k_8}{\longleftrightarrow}} HHpMK3 \tag{3}$$

$$HHpMK3 \xrightarrow{k_9} HHpp + MK3 \tag{4}$$

$$HHpp + MK3 \underset{k_{-10}}{\overset{k_{10}}{\longleftrightarrow}} HHppMK3 \tag{5}$$

$$HHppMK3 \xrightarrow{k_{11}} HHppp + MK3 \tag{6}$$

$$HHppp + MK3 \underset{k_{-12}}{\overset{k_{12}}{\longleftrightarrow}} HHpppMK3 \tag{7}$$

$$HHpppMK3 \xrightarrow{k_{13}} HHpppp + MK3 \tag{8}$$

$$HHpppp + PP5 \underset{k_{-14}}{\overset{k_{14}}{\longleftrightarrow}} HHppppPP5 \tag{9}$$

$$HHppppPP5 \xrightarrow{k_{15}} HHpppPP5 \tag{10}$$

$$HHpppPP5 \underset{k_{-16}}{\overset{k_{16}}{\longleftrightarrow}} HHppp + PP5 \tag{11}$$

$$HHppp + PP5 \underset{k_{-17}}{\overset{k_{17}}{\longleftrightarrow}} HHpppPP5S \tag{12}$$

$$HHpppPP5S \xrightarrow{k_{18}} HHppPP5 \tag{13}$$

$$HHppPP5 \underset{k_{-19}}{\overset{k_{19}}{\longleftrightarrow}} HHpp + PP5 \tag{14}$$

$$HHpp + PP5 \underset{k_{20}}{\overset{k_{20}}{\longleftrightarrow}} HHppPP5S \tag{15}$$

$$HHppPP5S \xrightarrow{k_{21}} HHpPP5 \tag{16}$$

$$HHpPP5 \underset{k_{-22}}{\overset{k_{22}}{\longleftrightarrow}} HHp + PP5 \tag{17}$$

$$HHp + PP5 \underset{k_{-23}}{\overset{k_{23}}{\longleftrightarrow}} HHpPP5S \tag{18}$$

$$HHpPP5S \xrightarrow{k_{24}} HHPP5 \tag{19}$$

$$HHPP5 \underset{k_{-25}}{\overset{k_{25}}{\longleftrightarrow}} HH + PP5 \tag{20}$$

$$R6 = k_6[HH][MK3] - k_{-6}[HHMK3] \quad (21)$$
$$R7 = k_7[HHMK3] \quad (22)$$
$$R8 = k_8[HHp][MK3] - k_{-8}[HHpMK3] \quad (23)$$
$$R9 = k_9[HHpMK3] \quad (24)$$
$$R10 = k_{10}[HHpp][MK3] - k_{-10}[HHppMK3] \quad (25)$$
$$R11 = k_{11}[HHppMK3] \quad (26)$$
$$R12 = k_{12}[HHppp][MK3] - k_{-12}[HHpppMK3] \quad (27)$$
$$R13 = k_{13}[HHpppMK3] \quad (28)$$
$$R14 = k_{14}[HHpppp][PP5] - k_{-14}[HHppppPP5] \quad (29)$$
$$R15 = k_{15}[HHppppPP5] \quad (30)$$
$$R16 = k_{16}[HHpppPP5] - k_{-16}[HHppp][PP5] \quad (31)$$
$$R17 = k_{17}[HHppp][PP5] - k_{-17}[HHpppPP5S] \quad (32)$$
$$R18 = k_{18}[HHpppPP5S] \quad (33)$$
$$R19 = k_{19}[HHppPP5] - k_{-19}[HHpp][PP5] \quad (34)$$
$$R20 = k_{23}[HHp][PP5] - h_{-23}[HHpPP5S] \quad (35)$$
$$R21 = k_{24}[HHpPP5S] \quad (36)$$
$$R22 = k_{11}[HHPP5] - h_{66}[PP5][HH] \quad (37)$$
$$R23 = k_{20}[HHpp][PP5] - k_{-20}[HHppPP5S] \quad (38)$$
$$R24 = k_{21}[HHppPP5S] \quad (39)$$
$$R25 = k_{22}[HHpPP5] - k_{-22}[HHp][PP5] \quad (40)$$

In the above equations, HH is the HSF13S:HSE complex; HH_p,HH_{pp},HH_{ppp},and HH_{pppp} are the mono, di, tri, and tetra-phosphorylated complex; MK3 is the kinase; PP5 is a phosphatase, a co-chaperone; PP5S is the activated phosphatase formed during the dephosphorylation; and the rest of the dynamical variables are the complexes of MK3 or PP5A.

The kinetic equation for the production of mRNA90 from tetra phosphorylated HHpppp is given as follows:

$$HHpppp \xrightarrow{k_{26}} mRNA90 + HH + 4Pi \quad (41)$$

The corresponding rate equation is:

$$R26 = k_{26}[HH_{pppp}] \quad (42)$$

Rate equations for the phosphorylation-dephosphorylation reactions of trimer HSF1-HSE complex, (HH)

$$\frac{d[HH]}{dt} = R5 - R6 + R26 \tag{43}$$

$$\frac{d[HHp]}{dt} = R7 - R8 + R21 - R20 \tag{44}$$

$$\frac{d[HHpp]}{dt} = R9 - R10 - R23 + R19 \tag{45}$$

$$\frac{d[HHppp]}{dt} = R11 - R12 - R17 + R16 \tag{46}$$

$$\frac{d[HHpppp]}{dt} = R13 - R14 - R26 \tag{47}$$

$$\frac{d[HHMK3]}{dt} = R6 - R7 \tag{48}$$

$$\frac{d[HHpMK3]}{dt} = R8 - R9 \tag{49}$$

$$\frac{d[HHppMK3]}{dt} = R10 - R11 \tag{50}$$

$$\frac{d[HHpppMK3]}{dt} = R12 - R13 \tag{51}$$

$$\frac{d[HHppppPP5]}{dt} = R14 - R15 \tag{52}$$

$$\frac{d[HHpppPP5]}{dt} = R15 - R16 \tag{53}$$

$$\frac{d[HHpppPP5S]}{dt} = R17 - R18 \tag{54}$$

$$\frac{d[HHppPP5]}{dt} = R18 - R19 \tag{55}$$

$$\frac{d[HHppPP5S]}{dt} = R23 - R24 \tag{56}$$

$$\frac{d[HHpPP5]}{dt} = R24 - R25 \tag{57}$$

$$\frac{d[HHpPP5S]}{dt} = R20 - R21 \tag{58}$$

$$\frac{d[HHPP5]}{dt} = R21 - R22 \tag{59}$$
