# Supplementary material for: A Detailed Modular Analysis of Heat-Shock Protein Dynamics under Acute and Chronic Stress and Its Implication in Anxiety Disorders
Source: PLoS One. 2012 Aug 22;7(8):e42958. doi: 10.1371/journal.pone.0042958 (PMC3425570; doi:10.1371/journal.pone.0042958)
Supplement: Text S3 — Module 3: Production of the open form of HSP90 from mRNA90 and kinetic equations for the ATP-assisted conversion of HSP90 from an open to a closed conformation. (PDF) [file pone.0042958.s003.pdf]

Text S3

Module 3: Production of the open form of HSP90 from mRNA90 and kinetic equations for the ATP-assisted conversion of HSP90 from an open to a closed conformation

We consider the production of mRNA90, $HSP90_{op}$, and the stepwise conformational changes from open to closed form of HSP90, and formation of the negative feedback loop as module 3, and the kinetic steps are given below:

$$mRNA90 \xrightarrow{k_{27}} HSP90_{op} \tag{1}$$

$$HSP90_{op} + ATP \underset{k_{-28}}{\overset{k_{28}}{\longleftrightarrow}} HSP90_{ATPBND} \tag{2}$$

$$HSP90_{ATPBND} \underset{k_{-29}}{\overset{k_{29}}{\longleftrightarrow}} HSP90_{I1} \tag{3}$$

$$HSP90_{I1} \underset{k_{-30}}{\overset{k_{30}}{\longleftrightarrow}} HSP90_{I2} \tag{4}$$

$$HSP90_{I2} \underset{k_{-31}}{\overset{k_{31}}{\longleftrightarrow}} HSP90_{CL} \tag{5}$$

$$HSP90_{CL} \xrightarrow{k_{32}} HSP90_{op} + ADP \tag{6}$$

$$HSP90_{CL} \xrightarrow{k_{33}} \phi \tag{7}$$

$HSP90_{op}$ is formed from the translation of the mRNA of HSP90 (mRNA90), which is in the open form. For the sake of parsimony, we consider here three reactions in a single step: (i) mRNA90 translation into $HSP90_{90}$, (ii) dilution due to cell division leading to the reduction of mRNA90 concentration, and finally, (iii) mRNA90 degradation. This could be expanded into three first order reactions, but as we do not want to increase the number of steps, we combined all the three steps into one reaction. Adenosine triphosphate (ATP) is assumed to be present in excess, and therefore this is not explicitly considered in the rate equations. $HSP90_{op}$ is the open conformation of HSP90; $HSP90_{ATPBND}$ is the ATP-bound HSP90, and $HSP90_{I1,I2}$ are the two intermediate forms of HSP90 before the closed form of $HSP90_{CL}$ is obtained. The fate of $HSP90_{CL}$ is three fold; either it converts to open form or degrades or sequesters with HSF1S to form a negative feedback loop that control its own production.

Rate equations for the conversion of HSP90 protein from an open to a closed conformation

$$\frac{d[mRNAP90]}{dt} = k_{26}[HH_{pppp}] - k_{27}[mRNA90] \tag{8}$$

$$\frac{d[HSP90_{op}]}{dt} = k_{27}[mRNA90] + k_{32}[HSP90_{CL}] + k_{-28}[HSP90_{ATPBND}] - k_{28}[HSP90_{op}] \tag{9}$$

$$\frac{d[HSP90_{ATPBND}]}{dt} = k_{28}[HSP90_{op}] + k_{-29}[HSP90_{I1}] - (k_{-29} + k_{-28})[HSP90_{ATPBND}] \tag{10}$$

$$\frac{d[HSP90_{I1}]}{dt} = k_{29}[HSP90_{ATPBND}] + k_{-30}[HSP90_{I2}] - (k_{30} + k_{-29})[HSP90_{I1}] \tag{11}$$

$$\frac{d[HSP90_{I2}]}{dt} = k_{30}[HSP90_{I1}] + k_{hs44}[HSP90_{CL}] - (k_{31} + k_{-30})[HSP90_{I2}] \tag{12}$$

$$\frac{d[HSP90_{CL}]}{dt} = k_{31}[HSP90_{I2}] + (k_{-31} + k_{32})[HSP90_{CL}] - k_{33}[HSP90_{CL}] + R2 - R1 \tag{13}$$

$$\frac{d[HHSP90_{CL}]}{dt} = R1 - R2 \tag{14}$$

where P_i's are the phosphates.
