# Supplementary material for: A Detailed Modular Analysis of Heat-Shock Protein Dynamics under Acute and Chronic Stress and Its Implication in Anxiety Disorders
Source: PLoS One. 2012 Aug 22;7(8):e42958. doi: 10.1371/journal.pone.0042958 (PMC3425570; doi:10.1371/journal.pone.0042958)
Supplement: Text S4 — Simulation of the full ordinary differential equation model. (PDF) [file pone.0042958.s004.pdf]

Text S4

1 Simulation of the full ordinary differential equation model

The equations of Modules 1-3 and the following conservation relations are taken together to perform the bifurcation analysis and the numerical integration of the ODE model.

The conservation relations are as follows:

$$MK3 = MK3_{total} - (HHMK3 + HHpMK3 + HHppMK3 + HHpppMK3) \quad (1)$$

$$PP5 = PP5_{total} - (HHppppPP5 + HHpppPP5S + HHpppPP5 + HHppPP5 + HHppPP5S + HHpPP5 + HHpPP5S + HHPP5) \quad (2)$$

$$HSF1 = HSF1_{total} - (HSF1S + HSF12S + HSF13S + HHSP90_{cl} + HH + HHp + HHpp + HHppp + HHpppp + HHMK3 + HHpMK3 + HHppMK3 + HHpppMK3 + HHppppPP5 + HHpppPP5 + HHppPP5 + HHppPP5S + HHpPP5 + HHpPP5S + HHPP5) \quad (3)$$

$$HSE = HSE_{TOT} - (HH + HHp + HHpp + HHppp + HHpppp + HHMK3 + HHpMK3 + HHppMK3 + HHpppMK3 + HHppppPP5 + HHpppPP5 + HHpppPP5S + HHppPP5 + HHppPP5S + HHpPP5 + HHpPP5S + HHPP5) \quad (4)$$
